# Ambient fine particulate pollution and daily morbidity of stroke in Chengdu, China

**DOI:** 10.1371/journal.pone.0206836

**Published:** 2018-11-06

**Authors:** Wei Zeng, Yingcong Zhang, Liang Wang, Yonglan Wei, Rong Lu, Jinjie Xia, Bing Chai, Xian Liang

**Affiliations:** 1 Chengdu Center for Diseases Control and Prevention, Chengdu, Sichuan, P.R. China; 2 Chengdu High-tech Zone Center for Diseases Control and Prevention, Chengdu, Sichuan, P.R. China; 3 Chengdu Tianfu New Area Center for Diseases Control and Prevention, Chengdu, Sichuan, P.R. China; Institute for Nutritional Sciences, CHINA

## Abstract

**Introduction:**

Association has been reported between ambient fine particulate matter (PM) and adverse outcomes of cerebrovascular events. However, it remains unclear that whether short-term exposure to PM relates to stroke and the lag of health effects. This triggers us to examine the relationship between PM and population stroke morbidity in Chengdu.

**Methods:**

The daily average concentration of atmospheric pollutants and meteorological factors and daily morbidity of stroke in Chengdu (2013–2015) were collected. Based on time series analysis-generalized additive models (GAM), single-pollutant, two-pollutant and multi-pollutant model were established. The effects of atmospheric PM_2.5_ (defined as PM less than 2.5μm in aerodynamic diameter), PMc(defined as PM less than 10μm and more than 2.5μm in aerodynamic diameter) and PM_10_ (defined as PM less than 10μm in aerodynamic diameter) concentration on the daily mortality of stroke were analyzed, respectively.

**Results:**

The three-year mean concentrations of PM_2.5_, PMc and PM_10_ for air pollutants were 75.9, 43.9 and 119.7 μg/m^3^, respectively. PM_2.5_ on the current day (lag0) and with a moving average of 0–1 days were significantly associated with the increasing risk of stroke morbidity, and PM_2.5_ with a lag of 0–1 days had greater association, whereas for PMc and PM_10_ there were no significant association observed. In our study, every 10μg/m^3^ increase of PM_2.5_ was associated with 0.69% percent change in stroke morbidity (95%CI: 0.01~1.38). For females, every 10μg/m^3^ increase of PM_2.5_ contributes to 0.80% percent change of onset. And for the group of age less than 65, we observed 0.78% higher risk every 10μg/m^3^ increase of PM_2.5_.

**Conclusions:**

These findings suggest that short-term exposure to PM_2.5_ within 1 day is associated with the onset of stroke, and the younger people (age<65) and females are more sensitive than older people and males.

## Introduction

With the rapid development of economy in the past twenty years, several serious regional air pollution incidents were reported, covering the North, East, Southeast and Southwest regions of China, involving more than 1.3 million square kilometers, a population of 850 million people affected. According to the air quality surveillance data in 2013 from Ministry of Environmental Protection of the People’s Republic of China, the substandard days of air quality in 74 cities exceeded 68.4% of the year, and the proportion of severe and serious polluted days reached 30.2%. Among them, PM_2.5_ exceeded the standard most seriously, with an average exceeding standard rate of 68.9% and a maximum daily average of 766μg/m^3^. The global burden of disease (GBD) statistics estimated that in 2010, about 1.2 million Chinese residents died in advance related to PM_2.5_ pollution, accounting for about 1/9 of the total number of deaths in China[1].

Many domestic and foreign epidemiological studies showed that the short-term and long-term exposure to PM_2.5_ may cause adverse effects on human health, to respiratory system and cardiovascular system, pulmonary function changes and its structure, the change of immune function, increase the incidence of cancer [2,3,4]. And the ambient PM from haze is associated with many health effects, causing multi-system damage [5]. Study by Lisabeth LD et al. observed associations between recent PM_2.5_ and O_3_ exposure and ischemic stroke risk even in the community with relatively low pollutant level [6]. In a previous meta-analysis, PM_2.5_ contributes to 1.4% higher cerebrovascular disease mortality per 10μg/m^3^ increase [7]. Increasing number of literature looked at the association between PM_2.5_ and the hospital mortality of cardiovascular and cerebrovascular diseases. In China, the researches on ambient fine particulates are mainly concentrated in economically developed areas such as Beijing, Shanghai and Guangdong [8,9,10]. There is a lack of research data for Sichuan, one of the four major basins, where the inversion layer often appears, and it is conducive to the formation and maintenance of haze. To our knowledge, only a few studies have investigated the short-term health effects of PM_2.5_ on hospital admissions or mortality of stroke. Moreover, lacking of the population-based monitoring onset data, few examined the effects of PM_2.5_ on morbidity of such diseases. In this study, we used time-series analysis method based on generalized additive model to explore the association of PM_2.5_ with daily stroke morbidity in southwest China.

## Material and methods

### Data collection

Sichuan basin is one of the four major areas with high occurrence of haze. Chengdu, the capital city of Sichuan Province, covers a total land area of 12,400 km^2^ and 14 million people, located in the southwest of China, lies on the border of the plateau in the west and the basin in the east. The surrounding higher-elevation land helps contain cold air. When warm air moves over cooler air, the atmosphere would warm with altitude. Cool air gets trapped near the surface. The inversion layer is conducive to the formation and maintenance of haze. Temperature inversions often form in Chengdu, which provides a natural environment for fog and haze.

In 2003, Chengdu launched a program to collect both chronic disease morbidity and mortality data, and set up the death reporting system and chronic non-communicable diseases (NCD) monitoring system at the Chengdu Center for Disease Control and Prevention. The system includes demographic and disease information, for example, age, gender, onset and death date, etc. In this study, gender, age, onset date of stroke, diagnosis, International Classification of Disease etc. were obtained from this system. 414 monitoring sites including all of 40 tertiary hospitals, 42 country-level hospitals and 332 community health service centers (CHSC) were set up in all kinds of medical institutions; each site is responsible for the collection of onset and death information of stroke in its hospital. The CHSC also collected the information in their jurisdictions. The CDC staff at the county and municipal level needed to verify the data to correct any wrong information and delete duplicate records. The use of the data is under authorization and government regulation. The use of the data for commercial purpose and other purpose that may harm the interest of patient is forbidden.

Stroke onsets were classified according to the standard of International Classification of Diseases (ICD-10) by their direct causes. In this study, stroke includes cerebral infarction, cerebral stroke and cerebral vascular accident (ICD-10 code: I63, I64). A second onset of stroke would be recorded if the patient is attacked by stroke again after 28 days. We examined the daily stroke onsets over 1,095 consecutive days from 2013 to 2015.

In 2012, China introduced PM_2.5_ into one of the indicators to measure air quality, and started to monitor the concentration of PM_2.5_ of 113 cities in 2013. Air pollution data from 2013 to 2015, including PM with PM_10_ and PM_2.5_, nitrogen dioxide (NO_2_), sulfur dioxide (SO_2_) were derived from 8 national monitoring stations provided by Chengdu Environmental Monitoring Station. Daily temperature, relative humidity, atmospheric pressure and wind speed data were obtained from Chengdu Meteorological Bureau.

### Statistical method

Daily data of stroke onsets, ambient fine particulate pollution concentration and meteorological variables were linked by date and, therefore, can be analyzed with a time-series design. Because morbidity of stoke were rare, we fit the following generalized additive model (GAM) with a quasi-Poisson link function to explore the association between PM and stoke:

$$\mathrm{Log}\;E(Y_{t})=\beta Z_{t}+\mathrm{factor}\left(DOW_{t}\right)+\mathrm{ns}\left(Time_{t},7\right)+\mathrm{ns}(Temp_{t},6)+\mathrm{ns}\left(Rh_{t},3\right)+\mathrm{intercept}$$

Where the subscript t refers to the day of the study; *E*(*Y*_*t*_) represents the daily count of stroke onsets; *Z*_*t*_ is the daily concentration of ambient fine particulate pollution, such as PM_2.5_, SO_2_ and NO_2_; *β* represents the log-relative risk of stoke morbidity associated with a unit increase of ambient fine particulate pollution; *ns* mean the natural spline function. We included per year for calendar time, day of the week (*DOW*) and meteorological variables, such as temperature (*Temp*_*t*_) and relative humidity (*Rh*_*t*_*)* in the regressions. Relative risks of stoke morbidity with a 10μg/m^3^ increase in air pollution concentration were calculated. Percentage change equals relative risk minus 1 and then multiplies by 100.

We chose the degrees of freedom for each meteorological factor based on its best prediction for air pollution levels. Using degrees of freedom which predict best for ambient fine particulate pollution levels is advantageous because they will produce unbiased or asymptotically unbiased estimates of the pollution log-relative risk. Specifically, we chose 7 *df* per year for calendar time. According to the previous study [11],6 *df* for the mean of temperature of the current day (Temp 0) and 3 *df* for the current day’s humidity (Humidity 0).

To know the linear result of different concentration of air pollutant effect on stroke morbidity, we used the current-day and up to 5 days before the outcome (lag0-lag5) and moving averages of 1-day, 2-day (lag 0–1, lag 0–2). We used the smoothing function (4 df) to explore the dose-response relationships between PM concentrations and the log-relative risk of stroke. Subgroup analysis were conducted according to gender group (males and females) and age group (<65 years and ≥65 years). In addition, NO_2_ and SO_2_ were adjusted to test whether the associations were still sensitive in two or multi-pollutant models. The sensitivity of the key findings was assessed in terms of the degrees of freedom in the natural spline function of time trends (6–9 per year).

All statistical analyses were performed using R Programming Language (V.3.0.2, R Development Core Team) using the NLME, MGCV, packages. All statistical tests were two-sided, statistical significance was defined as *p <* 0.05.

## Results

### Data description

Table 1 summarizes the descriptive statistics of daily data on morbidity, air pollutants concentration and meteorological conditions. From January 1st in 2013 to December 31st in 2015, a total of 84, 535 onsets of stroke were reported, of which 52% were males, and 69% were aged over 65 years old.

**Table 1:** Distribution of numbers of daily stroke onset, air pollutants concentration and meteorological conditions in Chengdu, China (2013–2015).

Variables	Mean ± SD	Minimum	Frequency distribution			Maximum
			25%	50%	75%	
**Number of daily stroke onset**	77.2±0.87	4	60	74	89	442
**Male**	40.4±14.4	4	31	39	47	182
**Female**	36.6±15.9	1	27	34	44	260
**Age<65**	23.9±9.3	0	18	23	29	120
**Age≥65**	53.2±21.2	3	40	50	62	322
**Air pollutants concentration**						
**PM**_**10**_ **(μg/m**^**3**^**)**	119.7±77.91	15.3	65.9	99.5	150.4	818.1
**PM**_**2.5**_ **(μg/m**^**3**^**)**	75.9±51.73	10.4	39.4	60.3	95.6	397.6
**PM**_**c**_ **(μg/m**^**3**^**)**	43.9±36.51	0.1	23.5	36.4	55.6	562.4
**NO**_**2**_ **(μg/m**^**3**^**)**	119.7±77.91	3.6	65.9	99.5	150.4	129.5
**SO**_**2**_ **(μg/m**^**3**^**)**	21.0±13.14	15.7	12.1	16.6	25.3	82.3
**Weather condition**						
**Relative humidity (%)**	75.4±9.84	41.0	69.6	76.2	82.4	97.0
**Temperature (°C)**	17.2±7.11	1.5	10.9	18.4	23.1	30.1
**Daily atmospheric pressure**(**kPa**)	951.8±7.38	-	945.8	951.4	957.6	-
**Daily wind speed (m/s)**	1.1±0.37	-	0.9	1.1	1.3	-

The daily mean concentration of air pollutants PM_2.5_, PM_C,_ PM_10_, NO_2_ and SO_2_ were 75.9, 43.9, 119.7, 53.4 and 21.0μg/m^3^, respectively. The daily mean temperature and relative humidity was 17.2°C and 75.4%, respectively. For PM_2.5_ daily concentration, 38% of the observing days exceeding the Grade II national standards of National Ambient Air Quality Standards set by Ministry of environmental protection of People's Republic of China, which is set as 75μg/m^3^. And for PM_10_, there were 276 days (25%) exceeding the Grade II national standards (150μg/m^3^). The daily average concentration of PM_2.5_ and PM_10_ were highest in the first 3 months of the year, and the lowest during July, August and September, which was basically consistent with the trend of daily morbidity in stroke.

Spearman correlation analysis between daily mean concentration of air pollutants and meteorological factors showed that the concentration of ambient PM_2.5_ had a strong positive correlation with PM_10_ (r_s_ = 0.96), followed by the correlation between PM_10_ and NO_2_, which showed a moderate correlation of 0.80 (r_s_ = 0.80). Results were presented as r_s_ for each pair of pollutants or meteorological factors (Table 2). The results suggested that it was necessary to adjust the influence of coexisting pollutants and meteorological factors in the analysis of the relationship between atmospheric PM2.5 and daily morbidity.

**Table 2:** Spearman correlation coefficients between air pollutants and meteorological factors.

Air pollutants and meteorological factors	PM_2.5_	PM_C_	PM_10_	SO_2_	NO_2_	Average temperature	Relative humidity
**PM**_**2.5**_	1	0.70*	0.96*	0.75*	0.78*	-0.42*	-0.20*
**PM**_**C**_		1	0.86*	0.53*	0.70*	0.20*	-0.35*
**PM**_**10**_			1	0.73*	0.80*	-0.38*	-0.28*
**SO**_**2**_				1	0.69*	-0.31*	-0.36*
**NO**_**2**_					1	-0.31*	-0.16*
**Average temperature**						1	-0.11*
**Relative humidity**							1

Note

* *P*<0.05.

### Associations between PM and stroke morbidity

**Fig 1:**
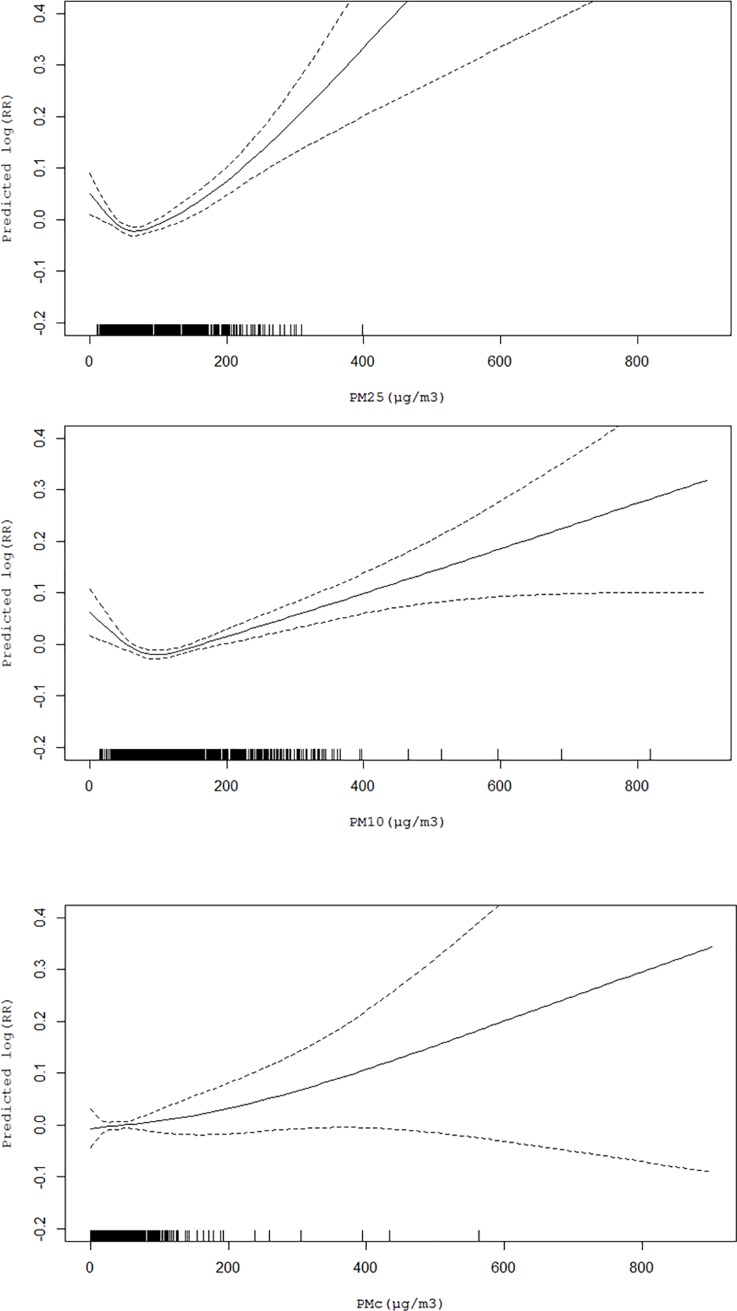
The smoothed plots of PM against the risk of morbidity of stroke.

The range of ambient fine particulate pollution exposures was wide and the concentrations were considerably high in the study. This pollution feature provides an opportunity to evaluate the shape of the exposure-response relationship across the full range of ambient fine particulate pollution exposures. There were clear dose–response relationships of PM _2.5_ concentration with stroke morbidity. The Fig 1 presents the pattern of stroke morbidity in relation to PM_2.5_, PM_C_ and PM_10_, the higher the concentration and the increased in the morbidity of stroke, with no threshold effects.

The X-axis is the current-day (lag 0 day) PM concentrations (μg/m^3^). Y-axis is the predicted log (relative risk (RR)), after adjusting for calendar time, day of the week, current-day temperature, and relative humidity.

### The associations of PM with stroke morbidity

According to the analysis of the lag effect of the single pollutant model, only PM_2.5_ was found significantly associated with elevated risk of stroke onsets while PM_C_ and PM_10_ were not (Table 3). The two-pollutant model and multi-pollutant model were fitted by choosing the most influential lag effect. We found that when NO_2_ and SO_2_ were introduced alone or at the same time, their effects on the daily morbidity of stroke disappeared with no statistical significance both in PM_2.5_ and PM_10_ under two-pollutant and multi-pollutant models (*P*>0.05).

**Table 3:** The percent change of daily stroke morbidity in every a 10μg/m^3^ increase in PM concentration under different pollutant models.

Pollutants	Model	Adjusting for pollutants	ER	95%CI	*P* value
**PM**_**2.5**_^**2**^	Single pollutant model	Null	0.60	0.01~1.19	0.04^1^
	two-pollutant model	SO_2_	0.63	-0.22~1.49	0.15
		NO_2_	-0.33	-1.29~0.64	0.50
	multi-pollutant model	SO_2_+NO_2_	-0.18	-1.17~0.82	0.72
**PM**_**C**_^**2**^	Single pollutant model	Null	0.26	-0.49–1.02	0.50
	two-pollutant model	SO_2_	0.1	-0.73–0.95	0.81
		NO_2_	-0.41	-1.3–0.49	0.37
	multi-pollutant model	SO_2_+NO_2_	-0.33	-1.23–0.58	0.47
**PM**_**10**_^**2**^	Single pollutant model	Null	0.29	-0.08–0.65	0.12
	two-pollutant model	SO_2_	-0.16	-0.66–0.34	0.53
		NO_2_	0.26	-0.19–0.71	0.26
	multi-pollutant model	SO_2_+NO_2_	-0.09	-0.61–0.43	0.73

Note

1. *P*<0.05.

2. The concentration of PM_2.5_ with a lag of 0–1 days, and PMc and PM_10_ on the current day were used in this model.

The percent change of daily stroke morbidity in every 10μg/m^3^ increase of PM_2.5_, PM_c_ and PM_10_ under different lag of days was computed. PM_2.5_ on the current day (lag0) and with a moving average of 0–1 days were significantly associated with the increasing risk of stroke morbidity, and PM_2.5_ with a lag of 0–1 days had greater association, whereas for PM_c_ and PM_10_ there was no significant association observed. Therefore we used the concentration of PM_2.5_ with a lag of 0–1 days, and PMc and PM_10_ on the current day to estimate the acute health effects on stroke. In our study, every 10μg/m^3^ increase of PM_2.5_ was associated with 0.69% percent change in stroke morbidity (95%CI: 0.01~1.38). For females, every 10μg/m^3^ increase of PM_2.5_ contributes to 0.80% percent change of stroke morbidity. And for the group of age less than 65, we observed 0.78% higher risk every 10μg/m^3^ increase of PM_2.5_ (Table 4).

**Table 4:** The percent change of daily stroke morbidity in every 10μg/m^3^ increase of PM_2.5_ to different genders and age groups under different lag of days.

Lag day	PM_2.5_				
	Total	Male	Female	Age<65	Age≥65
**lag0**	0.60(0.01~1.19) *	0.48(-0.10–1.07)	0.71(0.01–1.41)*	0.68(0.02–1.34)*	0.56(-0.07–1.2)
**lag1**	0.50(-0.13~1.13)	0.44(-0.18–1.05)	0.57(-0.16–1.32)	0.57(-0.13–1.27)	0.47(-0.20–1.14)
**lag2**	0.24(-0.37~0.86)	0.27(-0.33–0.88)	0.18(-0.54–0.91)	0.42(-0.26–1.11)	0.16(-0.49–0.82)
**lag3**	-0.10(-0.7~0.5)	-0.03(-0.61–0.56)	-0.24(-0.94–0.47)	-0.11(-0.77–0.55)	-0.10(-0.73–0.54)
**lag4**	0.03(-0.55~0.61)	0.01(-0.55–0.58)	0.05(-0.63–0.74)	0.06(-0.59–0.7)	0.02(-0.60–0.64)
**lag5**	-0.10(-0.67~0.48)	-0.10(-0.66–0.46)	-0.04(-0.71–0.64)	-0.06(-0.69–0.58)	-0.11(-0.72–0.5)
**lag0-1**	0.69(0.01~1.38)*	0.58(-0.09–1.25)	0.80(0–1.61)*	0.78(0.03–1.55)*	0.65(-0.08–1.38)
**lag0-2**	0.68(-0.08~1.45)	0.61(-0.13–1.35)	0.75(-0.14–1.65)	0.85(0.01–1.7)*	0.61(-0.2–1.42)

Note

*P<0.05

## Discussion

In 2012, the World Health Organization (WHO) reported that air pollutants could cause about 3,700,000 deaths worldwide every year, nearly 90% of them were from developing countries, and more than 20% of them died from myocardial infarction and stroke [12]. Even short time exposure to PM_2.5_ levels considered safe can increase the risk of stroke [13]. We obtained evidence in this study that PM_2.5_ concentration was associated with the increasing of stroke morbidity in Chengdu city, especially for females and those who age<65. Some studies pointed out that the health effect of PM_C_ could not be neglected. For example, significant association between PM_C_ and total mortality remained after adjusting for PM_2.5_ in Wang’s study, which indicated that PM_C_ may have effect on adverse health events[14]. And in another study conducted by Wang’s team, which specifically focused on the acute effects of coarse particle pollution on stroke mortality in six Chinese subtropical cities, also has proved that each 10 mg/m^3^ increase of PM_10_, PM_2.5_ and PM_C_ (lag03) was associated with an increase of 1.88% (95% CI: 1.37%, 2.39%), 3.07% (95% CI: 2.35%, 3.79%), and 5.72% (95%CI: 3.82%, 7.65%) in overall stroke mortality, respectively[15]. While associations between PM_C_ and PM_10_ and increased morbidity has not been proved in this study. At present, the researches on health effect of PM_2.5_ on stroke onset are still limited in China. Our estimates in Chengdu were similar in magnitude to two other PM_2.5_ mortality studies in Shenyang and Shanghai, China [16,17]. And our result also coordinated with a study in Guangzhou at the same period [18], which shows that in the single-pollutant model, the effects of current-day PM_2.5_ (RR = 1.0272, 95% CI: 1.0177–1.0368) exposure on stroke risk was statistically significant. The median level of PM_2.5_ over the years of 2013–2015 in Guangzhou was 41.0 μg/m^3^ (IQR, 27.0 to 60.0), which was lower than that in Chengdu (median: 60.3 μg/m^3^; IQR, 39.4 to 95.6).

According to previous studies, the effect of PM_2.5_ on stroke may vary with different study designs: time series and case crossover design. In this study, a 10μg/m^3^ increment in the lag of 0–1 days concentrations of PM_2.5_ corresponded to 0.69% (95%CI: 0.01~1.38) increase of total stroke morbidity, whereas for PM_C_ and PM_10_ there was no significant association observed. Based on the data of 75 cities of US, Dai et al. (2014) estimated a 1.76% (95% CI: 1.01, 2.52%) increase in stroke in association with a 10 mg/m^3^ increase in 2-day averaged PM_2.5_ concentration in time-series study [19]. While according to Villeneuve’s research [20], exposure to PM_2.5_ has no association with an increased risk of stroke in case crossover design. A meta-analysis conducted by Yu confirms that study design is a possible influence factor on the effect of PM_2.5_ on stroke onset [21].

At present, the pathophysiology mechanism of stroke caused by air pollution still lacks evidence. The possible mechanisms include thrombosis, inflammation and dysfunction of vascular endothelial cells. Atmospheric pollutants may promote circulation by increasing the fiber protein, C reactive protein and white blood cells, and thus induce systemic inflammation, and increase blood viscosity [22]. In addition, air pollutants may also cause damage to vasoconstrictor function, which can lead to abnormalities in the cardiovascular system, such as blood pressure and heart rate changes [23]. The difference in aerodynamics diameter of particles has different effects on human health. Larger particles, such as PM_10_, are more susceptible to respiratory system, but smaller particles or ultrafine particles are more easily inhaled into the blood, so they are more inclined to play a role in the cardiovascular and cerebrovascular system. This helps explain why PM_2.5_ has a significance correlation with the increase in stroke incidence.

In previous studies, age and gender are also an important factor in the impact of PM on stroke. Like other studies [24,25], we observed significant effect of PM_2.5_ stroke morbidity in female group; every 10μg/m^3^ increase of PM_2.5_ contributes to 0.80% percent change of stroke morbidity. Using the Women’s Health Initiative Observational Study [26], the investigators found a hazard ratio of 1.28 (95%CI: 1.02–1.61) for stroke associated with 10μg/m3 increases in PM_2.5_. Study by Kim and Hu [27] has found that PM deposition characteristics are different between males and females under controlled breathing conditions. Their measurement has also found that deposition in females is greater than that in males. The authors implicate in health risk assessment concerning inhaled particles that regional deposition enhancement in women may lead to a greater health risk. An experimental study of 50 persons [28] showed significant positive associations between personal PM_2.5_ exposure and oxidation products in females but not in males, which suggests that females possibly are more sensitive to airborne pollution than are males because they have fewer red blood cells and thus may be more sensitive to toxicological influences of air pollutants. In this study, the result indicates that females are more susceptible than males. This also may be explained by higher airway hyper responsiveness to oxidants, or relatively lower socioeconomic status [29].

Larrieu et al. studied stroke incidence and air pollutants in 8 cities in France [30]. No association was found between stroke onsets and air pollution levels. However, PM_10_ had greater impact on the daily number of hospitalizations of cardiovascular diseases among people who were older than 65 years old, indicating that older people may be more susceptible to air pollutants. As a contrast, our study has found that younger populations are more vulnerable when exposed to PM_2.5_. We observed 0.78% higher risk every 10μg/m^3^ increase of PM_2.5_ in the group of age<65. This result accorded with the ACS Study (American Cancer Society Study of Particulate Air Pollution and Mortality) that population of age<60 would experience 1.04 (95%CI: 1.00–1.09) times higher risk every 10μg/m^3^ increase of PM_2.5_ in all-cause mortality. The reasons for this difference of age group are unclear. The observed vulnerability for the group of age<65 may be explained by the increasingly air pollution serious in recent decades, and the younger generation suffer from that earlier in their life cycle. In addition, the possible explanation for the age differences seen in Chengdu was that older people pay more attention to prevent harm from dust events. For instance, older people are more inclined to stay home during high-concentration particulate days, but many younger people do not.

Several limitations should be noted in this study. Firstly, we have not searched for the influence of PM_2.5_, PM_C_ and and PM_10_ on subtypes of stroke (ischemic and hemorrhagic). It is possible that either ischemic stroke or hemorrhagic stroke is related with air pollution. Previous studies by Maheswaran R and Wellenius GA indicate that ischemic stroke has an association with air pollution [31,32]. And a meta-analysis on short-term effects of PM on stroke attack by Xiaobo Yu [17] also mentioned PM_2.5_ and PM_10_ were both associated with an increased risk of ischemic stroke, while for hemorrhagic no association was observed both for PM_2.5_ and PM_10_. However, study conducted in Guangzhou, China by Lin H [33] found there was significant association between PM pollution and hemorrhagic stroke mortality, but not ischemic stroke mortality. Secondly, as in most previous time-series studies, we averaged pollutant measurements across monitors within a city. This results in measurement error, which is difficult to quantify, especially in 2-pollutant models. Thirdly, we have not explored the relationship between a certain component from fine particles and stroke, which different component of particles may lead to diverse outcomes of cerebrovascular events. And fourthly, the subjectivity of physicians in the disease diagnosis among different hospitals may have influenced the effect estimates.

## Conclusion

In Chengdu, where growing industries have brought large amount of air pollution to environment, public awareness of multifarious effects of ambient PM on health issues is increasing. In our study, significant association has been found between stoke morbidity and elevated PM_2.5_ concentration, and younger people (age<65) and females might be more vulnerable to exposure to PM_2.5_ compared with older people and males. Our findings might be useful for the prevention of stroke onset by air pollution and may have implications for local policy makers working to improve the air quality. Furthermore, the association of PM_2.5_ and subtypes of stroke should be studied in future study.

## Supporting information

S1 Table The percent change of daily stroke morbidity in every a 10μg/m3 increase in fine PM concentration under different pollutant models. (DOCX)

S2 Table The percent change of daily stroke morbidity in every 10μg/m3 increase of PM2.5 to different genders and age groups under different lag of days. (DOCX)

S3 Table The number of missing data of each air monitoring station of 3 years. (DOCX)

S4 Table The sensitivity analysis by changing the df of the long-term trend. (DOCX)

S5 Table The raw data needed to replicate the findings of this study. (CSV)
