# Supplementary material for: Ambient fine particulate pollution and daily morbidity of stroke in Chengdu, China
Source: PLoS One. 2018 Nov 6;13(11):e0206836. doi: 10.1371/journal.pone.0206836 (PMC6219788; doi:10.1371/journal.pone.0206836)
Supplement: S1 Table — (DOCX) [file pone.0206836.s001.docx]

# Supporting information

**S1 Table The percent change of daily stroke morbidity in every a 10μg/m^3^ increase in fine PM concentration under different pollutant models**

| **Pollutants** | **Model** | **Adjusting for pollutants** | **ER** | **95%CI** | ***P*value** |
| --- | --- | --- | --- | --- | --- |
| **PM_2.5_** | Single pollutant model | Null | 0.60 | 0.01~1.19 | 0.04* |
|  | two-pollutant model | SO_2_ | 0.63 | -0.22~1.49 | 0.15 |
|  |  | NO_2_ | -0.33 | -1.29~0.64 | 0.50 |
|  | multi-pollutant model | SO_2_+NO_2_ | -0.18 | -1.17~0.82 | 0.72 |
| **PM_C_** | Single pollutant model | Null | 0.26 | -0.49-1.02 | 0.50 |
|  | two-pollutant model | SO_2_ | 0.1 | -0.73-0.95 | 0.81 |
|  |  | NO_2_ | -0.41 | -1.3-0.49 | 0.37 |
|  | multi-pollutant model | SO_2_+NO_2_ | -0.33 | -1.23-0.58 | 0.47 |
| **PM_10_** | Single pollutant model | Null | 0.29 | -0.08-0.65 | 0.12 |
|  | two-pollutant model | SO_2_ | -0.16 | -0.66-0.34 | 0.53 |
|  |  | NO_2_ | 0.26 | -0.19-0.71 | 0.26 |
|  | multi-pollutant model | SO_2_+NO_2_ | -0.09 | -0.61-0.43 | 0.73 |

Note:1. * *P*<0.05.

The degrees of freedom for each meteorological factor based on its best prediction for air pollution levels.

The concentration of PM_2.5_ with a lag of 0-1 days, and PM_c_ and PM_10_ on the current day were used in this model .
