# Supplementary material for: Ambient fine particulate pollution and daily morbidity of stroke in Chengdu, China
Source: PLoS One. 2018 Nov 6;13(11):e0206836. doi: 10.1371/journal.pone.0206836 (PMC6219788; doi:10.1371/journal.pone.0206836)
Supplement: S2 Table — (DOCX) [file pone.0206836.s002.docx]

**S2 Table The percent change of daily stroke morbidity in every 10μg/m^3^ increase of PM_2.5_ and PM_10_ to different genders and age groups under different lag of days**

| **Lag day** | **PM_2.5_** | | | | |
| --- | --- | --- | --- | --- | --- |
|  | **Total** | **Male** | **Female** | **Age<65** | **Age≥65** |
| **PM_2.5_** | | | | | |
| **lag0** | 0.60(0.01~1.19) ^*^ | 0.48(-0.10-1.07) | 0.71(0.01-1.41)^*^ | 0.68(0.02-1.34)^*^ | 0.56(-0.07-1.2) |
| **lag1** | 0.50(-0.13~1.13) | 0.44(-0.18-1.05) | 0.57(-0.16-1.32) | 0.57(-0.13-1.27) | 0.47(-0.20-1.14) |
| **lag2** | 0.24(-0.37~0.86) | 0.27(-0.33-0.88) | 0.18(-0.54-0.91) | 0.42(-0.26-1.11) | 0.16(-0.49-0.82) |
| **lag3** | -0.10(-0.7~0.5) | -0.03(-0.61-0.56) | -0.24(-0.94-0.47) | -0.11(-0.77-0.55) | -0.10(-0.73-0.54) |
| **lag4** | 0.03(-0.55~0.61) | 0.01(-0.55-0.58) | 0.05(-0.63-0.74) | 0.06(-0.59-0.7) | 0.02(-0.60-0.64) |
| **lag5** | -0.10(-0.67~0.48) | -0.10(-0.66-0.46) | -0.04(-0.71-0.64) | -0.06(-0.69-0.58) | -0.11(-0.72-0.5) |
| **lag0-1** | 0.69(0.01~1.38)^*^ | 0.58(-0.09-1.25) | 0.80(0-1.61)^*^ | 0.78(0.03-1.55)^*^ | 0.65(-0.08-1.38) |
| **lag0-2** | 0.68(-0.08~1.45) | 0.61(-0.13-1.35) | 0.75(-0.14-1.65) | 0.85(0.01-1.7)^*^ | 0.61(-0.2-1.42) |

Note: ^*^*P*<0.05

0-1 means the moving averages of current-day and previous-day concentrations of fine particulate pollution in the regressions.

0-2 means the moving averages of current-day and previous 2 day2 concentrations of fine particulate pollution in the regressions.
