# Supplementary material for: Ambient fine particulate pollution and daily morbidity of stroke in Chengdu, China
Source: PLoS One. 2018 Nov 6;13(11):e0206836. doi: 10.1371/journal.pone.0206836 (PMC6219788; doi:10.1371/journal.pone.0206836)
Supplement: S3 Table — (DOCX) [file pone.0206836.s003.docx]

**S3 Table The number of missing data of each air monitoring station of 3 years**

**We used the data from 8 national air monitoring stations in our city. The number of missing data from year 2013 to 2015 of each monitoring station is presented in table 1, which was replaced by the average value of existing data.**

| monitoring stations | PM10 | PM2.5 | SO2 | NO2 |
| --- | --- | --- | --- | --- |
| Caotang Temple | 9 | 6 | 7 | 6 |
| Jinquan | 13 | 31 | 37 | 27 |
| Liangjia Roadway | 11 | 13 | 15 | 21 |
| Lingyan Mountain | 28 | 36 | 21 | 18 |
| People's Park | 12 | 21 | 21 | 26 |
| Sanwayao | 3 | 19 | 23 | 7 |
| Shahepu | 2 | 19 | 24 | 19 |
| Shilidian | 3 | 12 | 8 | 10 |
| total | 81 | 157 | 156 | 134 |

We used the data from 8 national air monitoring stations in our city. The number of missing data from year 2013 to 2015 of each monitoring station is presented in table 1, which was replaced by the average value of existing data.
