# Supplementary material for: Ambient fine particulate pollution and daily morbidity of stroke in Chengdu, China
Source: PLoS One. 2018 Nov 6;13(11):e0206836. doi: 10.1371/journal.pone.0206836 (PMC6219788; doi:10.1371/journal.pone.0206836)
Supplement: S4 Table — (DOCX) [file pone.0206836.s004.docx]

**S4 Table The sensitivity analysis by changing the df of the long-term trend**

| DF | ER | 95%CI | P value |
| --- | --- | --- | --- |
| 3*6 | 0.51 | -0.07-1.09 | 0.09 |
| 3*7 | 0.59 | 0-1.19 | 0.05 |
| 3*8 | 0.62 | 0.02-1.22 | 0.04 |
| 3*9 | 0.67 | 0.07-1.28 | 0.03 |

Previous studies showed that the change of degree of freedom (df) in temperature and humidity has little impact on the value of health effect. Therefore we conduct the sensitivity analysis only by changing the df (6-9 per year) of the long-term trend. The results are listed in the following table. With the change of df in a considerable range, the effect value had little change, which suggests that the model is robust.
